# Enhancement of Solasodine Extracted from Fruits of *Solanum nigrum* L. by Microwave-Assisted Aqueous Two-Phase Extraction and Analysis by High-Performance Liquid Chromatography

**DOI:** 10.3390/molecules24122294

**Published:** 2019-06-21

**Authors:** Li Lin, Wen Yang, Xing Wei, Yi Wang, Li Zhang, Yunsong Zhang, Zhiming Zhang, Ying Zhao, Maojun Zhao

**Affiliations:** 1 College of Science, Sichuan Agricultural University, Yaan 625014, China; 14211@sicau.edu.cn (L.L.); Yangwen1611@163.com (W.Y.); weixing19960518@126.com (X.W.); 18728191338@163.com (Y.W.); zhangli@sicau.edu.cn (L.Z.); yaanyunsong@126.com (Y.Z.); zhaoying7767@163.com (Y.Z.); 2 Maize research institute, Sichuan Agricultural University, Chengdu 611130, China; zhzhang@sicau.edu.cn

**Keywords:** microwave-assisted aqueous two-phase extraction, Solasodine, alkaloid, response surface methodology, high-performance liquid chromatography

## Abstract

**Background:** Solasodine is a major bioactive ingredient in *Solanum nigrum* L. that has strong pharmacological characteristics. Therefore, the development of a simple and effective extraction method for obtaining solasodine is highly important. This study aims to provide a rapid and effective method for extracting solasodine from *Solanum nigrum* L. by microwave-assisted aqueous two-phase extraction (MAATPE). **Methods:** First, the high-performance liquid chromatography (HPLC) conditions were established for the detection of solasodine. Then, the aqueous two-phase system (ATPS) compositions were examined. On the basis of the results of single-factor experiments, for a better yield, response surface methodology (RSM) was used to optimize influential factors including the extraction temperature, extraction time and liquid-to-solid ratio. **Results:** The maximum extraction yield of 7.11 ± 0.08 mg/g was obtained at 44 °C, an extraction time of 15 min, and a liquid-to-solid ratio of 42:1 mL/g in the ATPS consisting of EtOH solvent, (NH_4_)_2_SO_4_, and water (28:16:56, *w*/*w*/*w*). The extraction yield of the alkaloid obtained using this method was markedly higher than those of microwave-assisted extraction (MAE) and ultrasonic-assisted extraction (UAE). **Conclusions:** In this work, solasodine was extracted by MAATPE for the first time and a high yield was obtained. MAATPE is a simple, rapid, and green technique for extraction from medical plants. Thus, the present study will enable the development of a feasible extraction method of active alkaloids from *Solanum nigrum* L.

## 1. Introduction

*Solanum nigrum* L. belongs to the Solanaceae family. As a typical traditional medicine in the Chinese pharmacopoeia, its leaves and fruits possess high medical value for treatments to clear heat, remove toxins, reduce swelling, and heal inflammation. Alkaloids are the major active constituents in this plant. In recent years, *Solanum nigrum* L. extracts, with antibacterial, antiviral, antioxidant, liver trauma treatment, and antineoplastic activities, have been extensively developed through a series of experiments, and considerable progress has been made [1,2,3,4,5]. Solasodine (in *Solanum nigrum* L.), the aglycone of steroidal alkaloids, can be obtained by hydrolysis and is a medically important component that is mainly used for antineoplastic drugs [6,7].

Solasodine is easily soluble in benzene, pyridine, and chloroform, soluble in ethanol, methanol, and acetone, and slightly soluble in water, and is closely connected to the cell wall in plants by aglycone. Conventional extraction methods, such as heat reflux extraction, Soxhlet extraction, and supercritical fluid extraction, have been developed to extract alkaloids from *Solanum nigrum* L. [8,9,10,11]. However, these processes suffer from obvious shortcomings, such as a high operating cost, poor extraction efficiency, long extraction time, and high usage of solvent in the products. Most recently, microwave-assisted extraction (MAE) has been widely applied to intensify the extraction and separation process [12] in the extraction of active substances from food, natural products, and traditional Chinese medicinal herbs. Microwave energy can enhance the penetration of the solvent into the matrix, expediting the release of bioactive compounds, which could significantly increase the extraction rate of the active ingredients from plants. The main advantages of MAE over the conventional extraction techniques are a short extraction time, smaller solvent consumption, and good selectivity [13,14,15].

An aqueous two-phase system (ATPS) can be spontaneously formed by mixing two water-soluble polymers or a water-soluble polymer and a salt aqueous solution at specific concentrations. Based on the differences in the compositions of the two phases, ATPS is divided into four types: polymer aqueous two-phase extraction [16], small molecule organic solvent aqueous two-phase extraction [17], surfactant aqueous two-phase extraction [18], and ionic liquid aqueous two-phase extraction [19]. Compared with other types of ATPS, the small molecular alcohol ATPS has outstanding advantages, such as a low cost, low viscosity, easy recovery, homogeneous phase extraction, and lack of phase emulsification. Therefore, aqueous two-phase extraction (ATPE) is considered to be a versatile and effective alternative to the conventional extraction methods. ATPE has been applied to the separation of compounds, such as the effective components of natural products [20,21,22], biological molecules [23,24,25], metal ions [26], and organic compounds [27]. Based on this technique, the novel microwave-assisted aqueous two-phase system extraction (MAATPE) method combines extraction and purification and offers some of the advantages of MAE and ATPE due to the demixing effect and microwave action, reduction of the extraction time, improved product yield, and increased purity of the extracted alkaloids [22,28]. Compared to ultrasound-assisted extraction (UAE) and other typical extraction methods, the MAATPE one-step extraction method is a promising and powerful alternative for the extraction and purification of alkaloids from *Solanum nigrum* L.

The application of MAATPE to the extraction of solasodine from *Solanum nigrum* L. has not been reported to date. The present study aimed to evaluate and maximize the potential and effectiveness of MAATPE as a rapid and effective method for the extraction of solasodine. After an initial screening, different ATPSs were tailored for the highest extraction efficiency, followed by the optimization of the extraction conditions carried out using the response surface methodology (RSM) to provide the most efficient process [29]. Moreover, to evaluate the feasibility and superiority of MAATPE, MAE and UAE methods, using an ethanol aqueous solution without salts, were investigated for comparison. Finally, high-performance liquid chromatography (HPLC) is known to be the most precise and sensitive detection method, and, therefore, HPLC was used to determine the efficiency of the solasodine extraction.

## 2. Results

### 2.1. Qualitative Analysis of Solasodine

**Figure 1:**
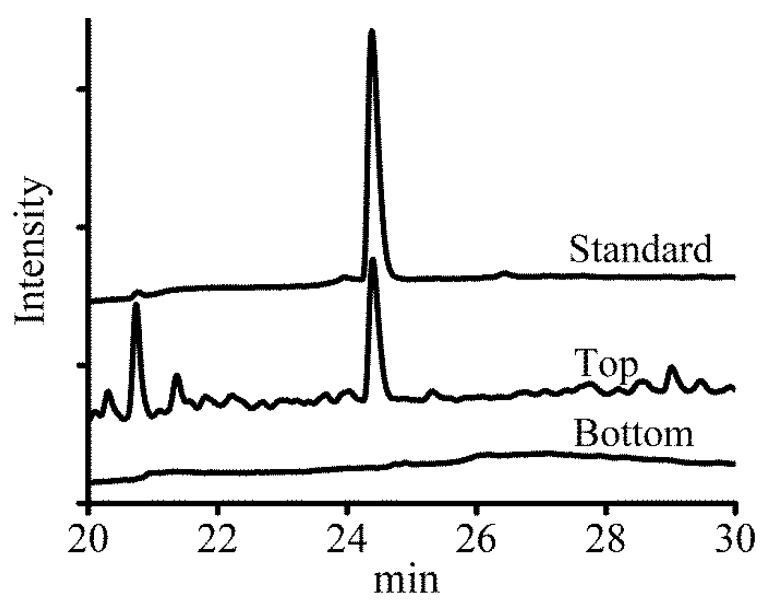
HPLC-UV chromatogram of solasodine from microwave-assisted aqueous two-phase system extraction (MAATPE).

As shown in Figure 1, the contents of the extracts from the top phase and bottom phase of the ATPS were determined by HPLC analysis and were compared with the pure standard of solasodine. Almost no alkaloids were present in the bottom phase. This is because alkaloids show good solubility in the organic solvents of the top phase [22].

### 2.2. Screening of the ATPS 

**Figure 2:**
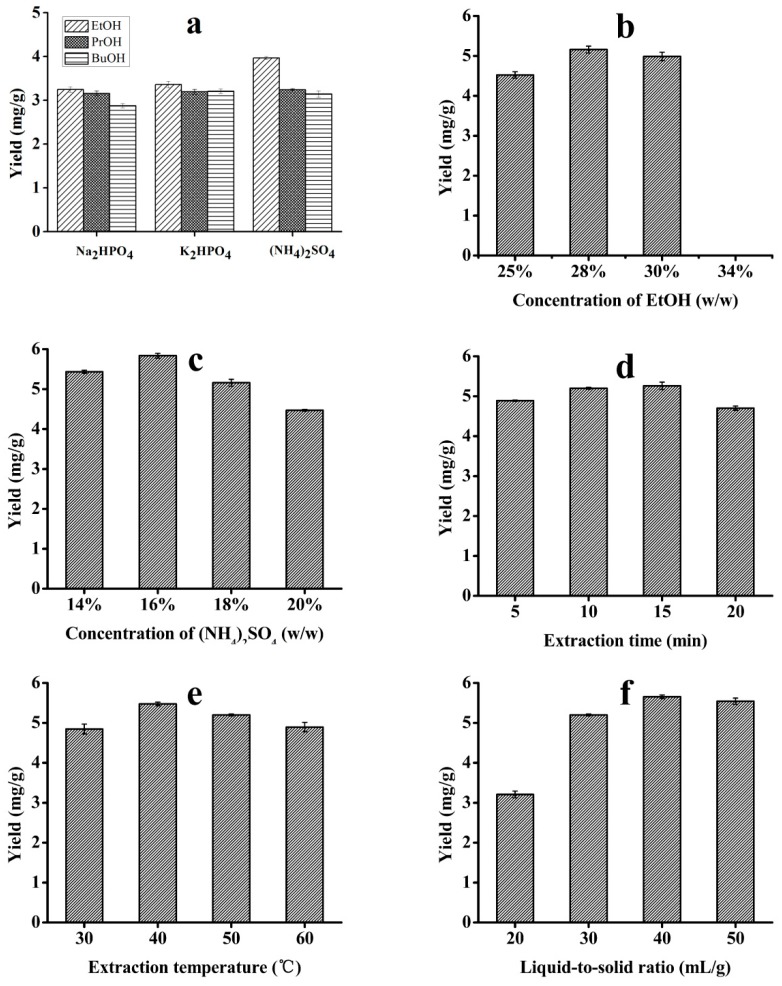
Different parameters of (**a**) type of aqueous two-phase system (ATPS), (**b**) EtOH concentration, (**c**) (NH_4_)_2_SO_4_ concentration, (**d**) ultrasonication time, (**e**) extraction temperature, and (**f**) liquid-to-solid ratio for the extraction of solasodine using MAATPE.

The effects of different alcohols (PEG, EtOH, n-PrOH, and n-BuOH) and salts ((NH_4_)_2_SO_4_, K_2_HPO_4_, Na_2_HPO_4_, Na_2_CO_3_, and Na_2_SO_4_) on the solasodine extraction were studied. Among these, the bottom phase was easily saturated and precipitated for Na_2_CO_3_ and Na_2_SO_4_ due to the narrow range of the ATPS formed by these salts. The PEG/salt results showed that the PEG removal was complicated due to the high viscosity of PEG. Based on the preliminary experimental results, alcohols (EtOH, n-PrOH, and n-BuOH) and salts ((NH_4_)_2_SO_4_, K_2_HPO_4_, and Na_2_HPO_4_) were employed for the further study of the extraction performance. Here, nine types of ATPS were investigated, displaying diverse extraction abilities. As shown in Figure 2a, EtOH/(NH_4_)_2_SO_4_ was employed for the further optimization of the extraction conditions. 

According to previous works [30,31], the composition of the ATPS lies within a small range of EtOH and (NH_4_)_2_SO_4_ concentrations. Thus, the effect of the change in the EtOH content from 25% to 34% (*w*/*w*) was first studied under the conditions of an extraction temperature of 50 °C, extraction time of 10 min, and (NH_4_)_2_SO_4_ concentration of 18% (*w*/*w*). The obtained results are presented in Figure 2b and show that the maximum extraction yield was obtained with an EtOH concentration of 28% (*w*/*w*). When 28% EtOH was used as the extraction solvent, the polarity of the solvent was close to the polarity of solasodine, resulting in a high yield of up to 5.14 mg/g. Interestingly, increasing the concentration of EtOH to 34% resulted in the partial solubility of (NH_4_)_2_SO_4_ in the ATPS due to the interaction between the polarity and the volume ratio of the top phase, so that the ATPS cannot be formed at a constant temperature. Therefore, 28% EtOH was used in the subsequent experiments.

To optimize the yield, the (NH_4_)_2_SO_4_ concentration was also investigated in the range of 14% to 20% (*w*/*w*), while the EtOH concentration was kept at 28% (*w*/*w*) and the other parameters were as described above. As shown in Figure 2c, the extraction yield increased from 5.44 mg/g to 5.83 mg/g as the (NH_4_)_2_SO_4_ concentration changed from 14% to 16% and then decreased with the increasing (NH_4_)_2_SO_4_ concentration for (NH_4_)_2_SO_4_ concentrations greater than 18%. Therefore, 16% (*w*/*w*) (NH_4_)_2_SO_4_ and 28% (*w*/*w*) EtOH were chosen as the optimal ATPS composition parameters.

### 2.3. Single Factor Experiment 

#### 2.3.1. Effect of Extraction Time

The effects of the extraction time on the extraction yield of solasodine were investigated for extraction times in the range of 5 to 20 min, while the liquid-to-solid ratio and temperature were kept at 30:1 and 50 °C, respectively. As shown in Figure 2d, increasing the time from 5 to 15 min had a positive effect on the yield, while a lower yield was obtained for an extraction time of 20 min. This may be because a sufficiently long time is necessary to release the active component from the herb cells, while too long a time leads to its degradation and decomposition. Thus, the extraction time of 15 min was used in the subsequent optimization of the extraction conditions.

#### 2.3.2. Effect of Extraction Temperature

Figure 2e shows the effects of the extraction temperature on the yield of solasodine at temperatures ranging from 30 to 60 °C, with the other parameters selected according to the results described above. It was found that a relatively high yield was obtained, with the extraction efficiency decreasing gradually with higher temperatures for temperatures greater than 40 °C. It was concluded that higher temperatures led to a decrease in the surface tension and an increase in the vapour pressure within the microbubbles [32]. Thus, the greater penetration of the solvents and the solubility of solasodine improved the yield, but an excessive temperature led to the degradation and decomposition of the solasodine. 

#### 2.3.3. Effect of Liquid-to-Solid Ratio

The liquid-to-solid ratio is an important factor that can influence the extraction efficiency because the yield of solasodine is related to the contact area of the solid and liquid. As depicted in Figure 2f, the liquid-to-solid ratio was set to 20:1, 30:1, 40:1, and 50:1, respectively. The results obtained using these ratios indicate that the extraction efficiency was enhanced with the increasing liquid-to-solid ratio and reached a peak value at the liquid-to-solid ratio of 40:1; this is because an excess of solvent allows the largest contact area and makes the liquid penetrate into the solid more easily. However, when the amount of solvent reached a certain value, the solasodine was completely dissolved and the yield of solasodine increased only slightly with any further increase in the liquid-to-solid ratio. In contrast, the extraction equilibrium can be easily reached with insufficient solvent, possibly leading to incomplete penetration and poor extraction yield [33]. Therefore, the liquid-to-solid ratio of 40:1 was chosen for the subsequent experiments.

### 2.4. Optimization of the Procedure by RSM

Based on the results of the single-factor experiments, three parameters, namely, temperature, time, and liquid-to-solid ratio, were chosen for a further study of the interactions between the various factors by the RSM approach based on the Box-Behnken design (BBD), which is a collection of mathematical and statistical techniques. Thus, 17 experiments (runs 1–17, 12 factorial points, and 5 central points) were run, and the experimental values obtained for the yield of solasodine are presented in Table 1. The response variables were fitted to a second-order polynomial model equation estimated by the RSM:

(1) $$Y=\beta_{0}+\sum_{i=1}^{3}\beta_{i}X_{i}+\sum_{i=1}^{3}\beta_{ii}X_{i}^{2}+\sum_{i=1}^{3}\sum_{j=i+1}^{3}\beta_{ij}X_{i}X_{j},$$

where Y is the response variable, which is the yield of solasodine, $X_{i}$ and $X_{j}$ are the independent variables affecting the response, and $\beta_{0},\;\beta_{i},\;\beta_{ii},\;\beta_{ij}$ are the regression coefficients of the intercept, linear, quadratic, and interaction terms, respectively.

The significance of each coefficient was determined in the regression model. An analysis of variance for the evaluation of the second-order model is presented in Table 2. The model *p*-value was used to evaluate the significance of each coefficient, which is necessary to understand the pattern of the interactions between the independent variables. The *p*-value of solasodine was much smaller than 0.0001, indicating that the corresponding coefficients were highly significant. The R^2^ parameter was 0.9675, showing that more than 96.75% of the experimental data were fitted by the model. These results reveal that the regression model is highly reliable.

Based on the quadratic model, the optimal values of the independent variables and the response variable for the solasodine extraction were calculated as follows: an extraction temperature (X_1_) of 44 °C, extraction time (X_2_) of 15 min, and liquid to-solid ratio (X_2_) of 42:1 and a maximum predicted value of 7.04 mg/g for the yield. Under these optimal extraction conditions, triplicate validation experiments were carried out, and the average obtained extraction yield of solasodine was 7.11 ± 0.08 mg/g. This value agrees fairly well with the predicted result, indicating that the proposed method can be applied to optimize the conditions of solasodine extraction.

### 2.5. Comparison of MAATPE with UAE and MAE

To further evaluate the extraction efficiency of MAATPE, two conventional methods, MAE and UAE, were employed for comparison, following the procedures described in previous reports [34,35], and the results are summarized in Table 3. The extraction yield of solasodine by MAATPE is dramatically higher than those for UAE and MAE, which were performed using aqueous ethanol as the extractant without salts with an extraction time of 30 min. The use of ATPS by adding (NH_4_)_2_SO_4_ to the ethanol-water significantly improved the extraction efficiency. Thus, the proposed MAATPE method is an effective approach for the extraction of solasodine.

## 3. Discussion

### 3.1. Effect of ATPS Composition and Concentration

The ATPS consisting of inorganic salts and small molecule alcohols are formed due to the competition between the salts and alcohols for the water molecules to form their associated hydrates [36]. Therefore, the solubility of the salts in water and the molecular weight of the alcohols determined the partition coefficients of the two phases in the ATPS. As shown in Figure 2a, the maximum extraction yield was obtained using EtOH/(NH_4_)_2_SO_4_ as the extracting agent, owing to its good layering effect, high solubility for the alkaloid components in the plant materials, and high stability [30]. For the same alcohol, (NH_4_)_2_SO_4_ has higher solubility in water and a stronger water molecule competitiveness, increasing the alcohol phase concentration and improving the yield of the target component. Similarly, the ATPS formed from EtOH achieved high yields in the three alcohols, which can be explained by the principle of similar compatibility. As for the ATPS concentration, based on the similarity and intermiscibility theory, the solute derived from the plants is easily dissolved when the polarities of the solvent and solute are similar [30]. These results also demonstrated that salts could improve the conductivity and microwave action in the MAATPE, while the volume of the top phase decreased with the increase in the (NH_4_)_2_SO_4_ concentration, leading to reductions in the amounts of the target constituents [22,30].

### 3.2. Response Surface Analysis

**Figure 3:**
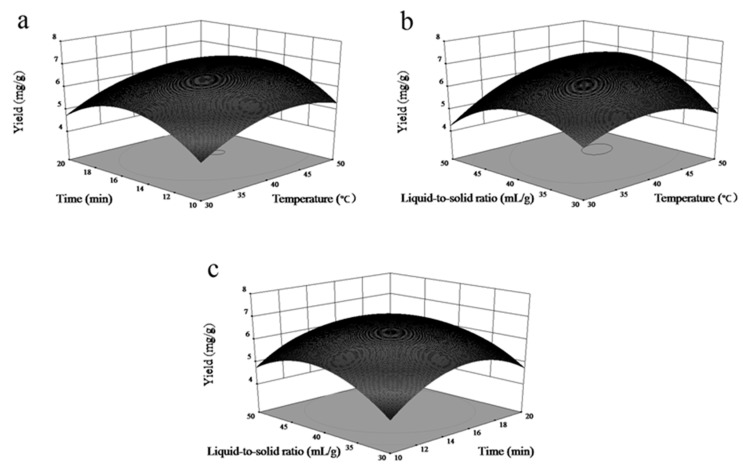
Response surface curves showing the effects of (**a**) time and temperature, (**b**) liquid-to-solid ratio and temperature, and (**c**) liquid-to-solid ratio and time on the extraction efficiency.

For a better understanding of the effects of each of the extraction factors and the optimal conditions for obtaining the maximum extraction yield, three three-dimensional profiles were plotted for analysing the interactions of the various process factors using Design-Expert 8.0 software, as shown in Figure 3. The three-dimensional profiles show how the three pairs of extraction parameters affect the extraction yield of solasodine. Figure 3a–c presents the combined effects of temperature (X_1_) and time (X_2_), temperature (X_1_) and liquid-to-solid ratio (X_3_), and time (X_2_) and liquid-to-solid ratio (X_3_), respectively. All three surfaces are top-convex with a maximum point in the centre of the experimental domain, indicating that the ranges of the factors were chosen correctly.

Figure 3a shows that the solasodine yield improved with increases in the temperature and time, as indicated by the positive coefficients (Table 4), while the negative interaction between X_1_ and X_2_ produced maximum values at temperatures higher than 40 °C and times longer than 15 min. According to the results presented in Figure 3b, similar conclusions can be reached based on the positive coefficients of X_1_, X_3_, and X_1_X_3_; therefore, a liquid-to-solid ratio higher than 30:1 is a better choice in the optimization. Figure 3c also shows the presence of significant interactions between X_2_ and X_3_.

## 4. Experimental

The minimum standards of reporting checklist contains details of the experimental design, statistics, and resources used in this study.

### 4.1. Chemicals and Plant Material

The dried herb fruit of *Solanum nigrum* L. was purchased from Baicaofang Co., Ltd. (Hebei, China). The samples were powdered, sieved (40 mesh), and placed in a desiccator at room temperature. Solasodine (purity ≥ 97%) was purchased from Chengdu Must Bio-Technology Co., Ltd. (Chengdu, China). HPLC grade acetonitrile was purchased from Thermo Fisher Scientific (Shanghai, China) and used in the HPLC analysis. Ammonium sulphate, anhydrous ethanol, and other chemicals of analytical grade were purchased from Chengdu Kelon Science Co., Ltd. (Chengdu, China).

### 4.2. Instruments and Analytical Methods

MAATPE experiments were carried out using a microwave extraction system (MAS-I) purchased from Sineo Microwave Chemistry Technology Co., Ltd. (Shanghai, China). The HPLC (LC-20A) was purchased from Shimadzu Corp. (Kyoto, Japan). The yield of solasodine in the extracts was determined using an Agilent C18 Column (250 mm × 4.6 mm, 5 µm, Santa Clara, CA, USA) at 40 °C with a UV detector at 210 nm. The injection volume was 10 µL, and gradient elution was performed at a flow rate of 1 mL/min over 50 min. The mobile phase, composed of solution A (0.1% phosphoric acid) and solution B (acetonitrile), was delivered as follows: 0 min, 10% (B); 30 min, 60% (B); 35 min, 90% (B); 40 min, 90% (B); 45 min, 10% (B); and 50 min, stop. 

### 4.3. Extraction Procedure

#### 4.3.1. Preparation of the Aqueous Two-Phase System 

According to a method reported in a previous study [21], different solvents and salts were employed for the formation of ATPS under particular mixing ratios. An appropriate amount of the inorganic salt was dissolved in deionized water and was then mixed with a certain volume of alcohol using a vortex stirrer. The extraction agent was obtained when the mixture showed two-phase separation.

#### 4.3.2. MAATPE Procedure 

The optimal extraction conditions are described as an example: the herb (0.71 g) powders and ATPS (30 mL) solvents (EtOH/(NH_4_)_2_SO_4_, 28:16, *w*/*w*) were added to a flask that was then placed in a microwave extraction system equipped with a cooling tube. The extraction was performed at 44 °C for 15 min, and the microwave power was set at 500 W in all of the related experiments, unless otherwise indicated. After cooling to room temperature, the extract solution was filtered to remove the herb residues, and the filtrate was held in a separatory funnel to allow phase separation. Then, the top phase was separated and concentrated to obtain the residues that were used in the subsequent hydrolytic reaction without any purification.

#### 4.3.3. Hydrolytic Procedure 

**Figure 4:**
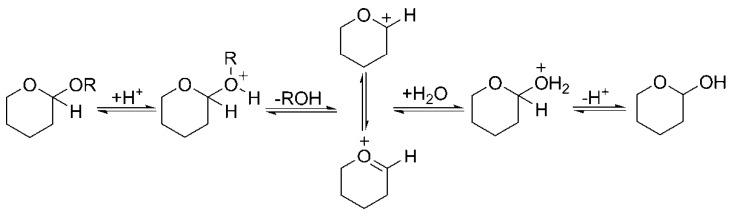
Hydrolysis mechanism of steroidal alkaloids, where R represents glycosyl.

The glucoside bond is generally due to aldolization, and in the hydrolysis mechanism, the chemical bonds between aglycone and glycosyl react easily and are broken under acidic conditions [7], as shown in Figure 4. Thus, the hydrolytic procedures were carried out as follows: the concentration derived from MAATPE was mixed with an HCl solution (20 mL, 2 mol/L) and anhydrous ethanol (20 mL). The hydrolysis temperature was kept at 100 °C for 2 h, and then the mixture was cooled. The acidity of the mixture was adjusted to pH = 10~11 to obtain free solasodine [37]. The solvent was removed by the vacuum-rotary evaporation procedure to obtain the residues. Subsequently, desalination was performed by adding methanol (10 mL) and centrifuging at room temperature for 1 min. Then, the supernatant (5 mL) was removed and diluted with methanol to 25 mL for detection. For reproducibility, the results reported for the solasodine extraction efficiency were the averages of three repeated trials (*n* = 3).

### 4.4. Optimization of Extraction Conditions 

The types of ATPS were screened in our initial experiments, together with four other main variables that affect the extraction efficiency, namely, the ATPS solvent concentration, extraction time, temperature, and liquid-to-solid ratio. Thus, the extraction conditions of MAATPE were first improved using single-factor experiments because the concentration of the solvent in the ATPS is small and was difficult to control in the RSM. Then, the RSM was applied to optimize the experimental conditions, including the extraction temperature, extraction time, and liquid-to-solid ratio. Design-Expert 8.0.6 software (Stat-Ease, Inc., Minneapolis, MN, USA) was employed not only for the experimental design but also for statistical analysis and regression modelling. The details of the RSM procedure are provided in Table 1. The parameters were screened based on the results of the single-factor experiments.

### 4.5. Conventional Extraction Methods

To compare the extraction efficiency of MAATPE to those of the conventional techniques, UAE and MAE were carried out at the optimized conditions determined in previous studies reported in the literature [34,35]. The UAE procedure at the optimal conditions was as follows: the sample powders (1.0 g) were mixed with aqueous ethanol (30 mL, 9:1, *v*/*v*) and extracted for 30 min using an ultrasonic generator (SB-600DTD, Scientz Biotechnology Co., Ltd., Ningbo, China). The MAE experiments were performed using the microwave device described above. The sample powders (1.0 g) were added to the ethanol aqueous solution (20 mL, 3:2, *v*/*v*) and extracted for 60 min. After extraction, the mixture was filtrated and the top phase was removed for analysis. The hydrolytic procedure was the same as that described in Section 2.3.3.

### 4.6. Statistical Analysis

All the experiments were carried out in triplicate. Design-Expert 8.0.6 software was employed not only for the experimental design, but also for statistical analysis and regression modelling. The Student’s t-test permitted the checking of the statistical significance of the regression coefficient, and Fisher’s F-test determined the second-order model equation at a probability (*p*) of 0.001, 0.01 or 0.05.

## 5. Conclusions

In the present study, MMAATPE was firstly introduced for improving the extraction yield of Solasodine from fruits of *Solanum nigrum* L., integrating MAE with ATPE into a one-step procedure and provided a rapid effective method for extracting Solasodine. Several factors were evaluated for the selection of the suitable ATPS and extraction methods by means of single factor experiment and RSM. The results indicated that the ATPS consisting of EtOH solvent, (NH_4_)_2_SO_4_ and water (28:16:56, *w/w/w*) provided superior extraction yields with less time consumption compared with the traditional method. Thus, this approach will enable the development of a feasible extraction method of alkaloid activities from fruits of *Solanum nigrum* L. In summary, MAATPE exhibits a simple, rapid, and green technique for the extraction in medical plants.

## Figures and Tables

**Table 1:** The experimental results of the Box-Behnken Design: X_1_, X_2_ and X_3_ are temperature, time, and liquid-to-solid ratio, respectively.

Run	Factors			Yield (mg/g)
	X_1_ (°C)	X_2_ (min)	X_3_ (mL/g)	
1	30	20	40	4.76
2	50	15	30	4.72
3	30	15	30	4.65
4	50	10	40	5.28
5	40	10	30	4.21
6	50	20	40	5.55
7	50	15	50	6.50
8	40	15	40	6.92
9	40	15	40	6.92
10	40	20	50	4.75
11	40	10	50	4.55
12	40	15	40	6.92
13	40	15	40	6.92
14	40	20	30	4.94
15	30	15	50	4.35
16	30	10	40	4.40
17	40	15	40	6.92

**Table 2:** Analysis of variance (ANOVA) for the second-order response surface model.

Source	Sum of Squares	Degrees of Freedom	Mean Square	F-Value	*p*-Value	Remarks
Model	18.71	9	2.08	53.87	<0.0001	significant
A(temperature)	1.89	1	1.89	49.01	0.0002	significant
B(time)	0.3	1	0.3	7.88	0.262	
C(liquid-to-solid ratio)	0.33	1	0.33	8.6	0.219	
AB	0.002	1	0.002	0.052	0.8254	
AC	1.08	1	1.08	28.02	0.0011	significant
BC	0.07	1	0.07	1.82	0.2194	
A2	2.31	1	2.31	59.74	0.0001	significant
B2	5.89	1	5.89	152.54	<0.0001	significant
C2	5.33	1	5.33	138.07	<0.0001	significant
Residual	0.27	7	0.039			
Lack of fit	0.27	3	0.09			
Pure error	0	4	0			
Cor total	18.98	16				

**Table 3:** Comparison of MAATPE with conventional methods with regard to the extraction yield of solasodine.

Method	Solvent	Time (min)	Tem. (°C)	Liquid-to-Solid Ratio (mL/g)	Yield (mg/g)
UAE	EtOH/water	30	25	30:1	3.39
MAE	EtOH/water	60	30	20:1	3.36
MAATPE	ATPS	15	44	44:1	7.11

**Table 4:** Estimated regression coefficients of the fitted second-order polynomial equation.

Factors	Coefficient	Df	Standard Error	95% Low	95% High	CL VIF
intercept	6.92	11	0.088	6.71	7.13	1
A- temp.	0.49	1	0.069	0.32	0.65	1
B- time	0.2	1	0.069	0.031	0.36	1
C- liquid-to-solid ratio	0.2	1	0.069	0.04	0.37	1
AB	−0.023	1	0.098	−0.25	0.21	1
AC	0.52	1	0.098	0.29	0.75	1
BC	−0.13	1	0.098	−0.36	0.1	1
A2	−0.74	1	0.096	−0.97	−0.51	1
B2	−1.18	1	0.096	−1.41	−0.96	1.01
C2	−1.12	1	0.096	−1.35	−0.9	1.01
